# Effects on growth performance and immunity of *Monopterus albus* after high temperature stress

**DOI:** 10.3389/fphys.2024.1397818

**Published:** 2024-04-24

**Authors:** Yifan Mao, Weiwei Lv, Weiwei Huang, Quan Yuan, Hang Yang, Wenzong Zhou, Mingyou Li

**Affiliations:** ^1^ Key Laboratory of Integrated Rice-Fish Farming, Ministry of Agriculture and Rural Affairs, Shanghai Ocean University, Shanghai, China; ^2^ Eco-Environmental Protection Research Institute, Shanghai Academy of Agricultural Sciences, Shanghai, China

**Keywords:** *Monopterus albus*, high temperature stress, immunity, liver, intestinal microorganisms

## Abstract

To investigate the impact of the effect of high temperature stimulation on *Monopterus albus* larvae after a certain period of time, five experimental groups were established at different temperatures. Then, the *M. albus* under high temperature stress was fed at 30°C for 70 days. After that, the growth index of the *M. albus* was counted and analyzed. In terms of growth index, high temperature stress had significant effects on FCR, FBW, WGR, and SGR of *M. albus* (*p <* 0.05). The SR increased after being stimulated by temperature (*p <* 0.1). The study revealed that liver cells of *M. albus* were harmed by elevated temperatures of 36°C and 38°C. In the experimental group, the activities of digestive enzymes changed in the same trend, reaching the highest point in the 32°C group and then decreasing, and the AMS activity in the 38°C group was significantly different from that in the 30°C group (*p* < 0.05). The activities of antioxidase in liver reached the highest at 34°C, which was significantly different from those at 30°C (*p* < 0.05). In addition, the expression levels of *TLR1*, *C3*, *TNF-α*, and other genes increased in the experimental group, reaching the highest point at 34°C, and the expression level of the *IL-1β* gene reached the highest point at 32°C, which was significantly different from that at 30°C (*p* < 0.05). However, the expression level of the *IRAK3* gene decreased in the experimental group and reached its lowest point at 34°C (*p* < 0.05). The expression level of the *HSP90α* gene increased with the highest temperature stimulus and reached its highest point at 38°C (*p* < 0.05). In the α diversity index of intestinal microorganisms in the experimental group, the observed species, Shannon, and Chao1 indexes in the 34°C group were the highest (*p* < 0.05), and β diversity analysis revealed that the intestinal microbial community in the experimental group was separated after high temperature stimulation. At the phylum level, the three dominant flora are *Proteus*, *Firmicutes*, and *Bacteroides*. *Bacteroides* and *Macrococcus* abundance increased at the genus level, but *Vibrio* and *Aeromonas* abundance decreased. To sum up, appropriate high-temperature stress can enhance the immunity and adaptability of *M. albus*. These results show that the high temperature stimulation of 32°C–34°C is beneficial to the industrial culture of *M. albus*.

## 1 Introduction

In recent years, with climate warming and global temperatures rising, the impact of high temperature stress on aquatic organisms has become increasingly prominent. A fish is a vertebrate whose immune system lags behind that of mammals, and its immune function is affected by environmental factors to some extent, among which temperature is one of the main factors. Extreme weather and precipitation alter temperature, salinity, and dissolved oxygen levels, creating stressors that impact the physiological wellbeing of aquatic species. These changes subsequently influence the health of cultured fish through hosts and/or infectious sources (Reid et al., 2019; Mugwanya et al., 2022).

Researchers found that moderate temperature rise enhances the immune defense ability of salmon by promoting the expression of immune-related genes (Gruli Barbosa et al., 2020). Proper temperature control can influence how the transcriptome responds to a virus and highlight the significant function of temperature in regulating the fish immune response (Hori et al., 2012). Further research shows that high temperature stimulation can promote heat shock response, apoptosis, and the expression of immune defense-related genes (Jeyachandran et al., 2023) which is especially obvious in the classical activation of the complement system (Beemelmanns et al., 2021). As a key part of teleosts’ natural defenses, the classical complement system can kill pathogens that get inside through LZM (Bai et al., 2022). In addition, stimulation at high temperatures can also trigger the inflammatory reaction of animals at variable temperatures, which is a key mechanism to deal with virus infection (Sanhueza et al., 2021). Dixon et al. (2016)’s research found that the survival rate of infectious hematopoietic necrosis virus (IHNV) in rainbow trout tissues was inversely proportional to temperature, emphasizing the influence of temperature on the interaction between pathogen survival and host immune response.

Environmental temperature significantly influences the microbial makeup and function of vertebrates that experience fluctuating temperatures (Fontaine et al., 2018), which is especially remarkable in fish because their immune response can be changed through the regulation of intestinal microbial flora (Sun et al., 2021). Compared with land animals, fish are more susceptible to continuous change in the environment, which may interfere with the stability of microbial communities (Butt and Volkoff, 2019). High temperature stress can result in damage to intestinal and accumulation of fat, reduced development rate, and decreased antioxidant capacity. The gut bacteria may contribute to these effects (He et al., 2019).

However, the stress process at too high a temperature may also have some side effects. For example, it has been found that the development of young pomfret ovata will be seriously affected in a high-temperature environment, leading to skeletal deformity (Yang et al., 2016). Under extreme temperature events, fish will use more energy to restore internal balance. Specifically, Fish might have to reallocate energy previously devoted to facilitating growth, maturation, immune functions, and reproductive activities towards repairing cells in injured tissues. This shift in energy usage could ultimately result in reduced growth efficiency and weakened immune responses (Islam et al., 2022). In addition, too high a temperature may also impair the immune system of fish, inhibit its immune function, and increase its risk of infection with pathogens.

When temperatures surpass the fish’s ideal living conditions, enzymatic activities tend to decelerate owing to the denaturation of enzymes (Strzelczak et al., 2021), especially the activities of various energy metabolism-related enzymes (such as alanine aminotransferase) and digestive enzymes (such as AMS) (Mazumder et al., 2018), and the decline of their activities will directly affect the digestion and absorption capacity of fish. In addition, high temperatures will also alter the pH value and ion concentration of the intestine, further inhibiting the activity of enzymes (Solovyev and Izvekova, 2016).

Moderate environmental pressure can promote the adaptability of fish. Temperature stress can activate and enhance the immune system of fish, improve its ability to adapt to environmental changes, and resist pathogen invasion, especially for *Monopterus albus* (*M. albus*), a wide-temperature fish. This mechanism promotes the functional regulation and promotion of the fish immune system through moderate stimulation and then enhances its defense ability against pathogens. For example, studies on *Paralichthys olivaceus* and *Oncorhynchus mykiss* show that Fish that are pre-exposed to elevated temperatures can enhance their immune response and boost their resistance to high temperatures, provided that these temperatures are marginally below their critical threshold (Golovanova et al., 2013; Li et al., 2024). Precisely controlling temperature stress can significantly improve the immune defense capability of fish.

*M. albus* is a common benthic fish in lakes, rivers, ditches, and rice fields. *M. albus* can be found in subtropical and tropical monsoon climate areas, from Java Island to the Liaohe River Basin, and can tolerate temperatures from 0°C to 40°C. Environmental temperature closely influences its growth and immune status. In 2022, the total output of *M. albus* in China was 334,251 tons, of which the output of Hubei Province accounted for 46% (“2022 China Fishery Statistics Yearbook,” 2023), and the output of *M. albus* in 2020 also has 313,790 tons, and the proportion of Hubei Province remained unchanged. Compared with 386,137 tons in 2017, the total output of *M. albus* decreased but remained at the same order of magnitude. Since China exceeded 300,000 tons in output in 2012, the culture technology of *M. albus* has not advanced, leaving the culture industry of *M. albus* in a bottleneck state.

Artificial propagation, opening, and high mortality of *M. albus* are three major problems faced by *M. albus* culture, which seriously restrict the development of the *M. albus* industry. The artificial propagation technology of *M. albus* has some defects, such as affecting the survival rate of parents, a low fertilization rate, a low hatching rate, and a low fry survival rate. At present, the main fry of the *M. albus* industry comes from capture in the wild, but in the wild environment, *M. albus* seedlings may carry some germs or viruses (Ou et al., 2013; Xia et al., 2019), which will explode rapidly in high-density artificial culture of *M. albus*, resulting in a large number of fish deaths. As a result, in the process of raising *M. albus*, farmers will choose the method of high temperature stimulation to reduce fry mortality. This study aims to examine the physiological and biochemical reactions of *M. albus* following acute high temperature stress at various temperatures during seedling development. It also seeks to assess the impact of different levels of high temperature stress on the immunity and growth of *M. albus*.

## 2 Materials and methods

### 2.1 Ethical statement

Animal experiments were carried out following the protocols described in the “Guide to Experimental Animals” issued by the Ministry of Science and Technology in China.

### 2.2 Experimental materials

The samples used in this study are from the Shanghai Academy of Agricultural Sciences. Prior to formal breeding trials, in order to ensure that the *M. albus* is able to adapt to the specific environment and conditions required for the experiment, all *M. albus* are fed in a 1.0 × 1.0 × 1.0 m cement pond. We used the natural ingredients that *M. albus* likes, namely, earthworms. The earthworm slurry was used as an attractant to make *M. albus* seedlings adapt to artificial feeding. A mixture of fish meal and water with a ratio of 3:7 was used as the main nutrient source. In the first week, they were mixed together in a ratio of 1:1 to make a paste-like open mixture, which was easily accepted by *M. albus* to initially stimulate the eating behavior of *M. albus*. In the next week, the proportion of earthworm slurry in the mixture gradually decreased to prevent the sudden change of feed from causing discomfort to *M. albus*. Until the feed used in the experiment was completely replaced by fish meal, this ensured that all the related *M. albus* smoothly transitioned to the standardized fish feed used in the experiment. At the end of the domestication phase, we randomly chose 150 healthy *M. albus* from the group as a whole. These *M. albus* were close together and had an average weight of 12.5 ± 0.7 g. They were then put into five trial groups and did so three times in each group. In order to maintain water quality conditions suitable for the growth of *M. albus*, exposure measures are used to replace new water daily, at about one-third of the total amount of water per replacement (NH^4+^-N < 0.5 mg/L, soluble oxygen ≥55.8 mg/L, and PH 7.3 ± 0.2). *M. albus* is bred using commodity feed (Hubei Hui Biotechnology Co., Ltd.). Artificial feeding at 16:00 every day is controlled in proportion to 4 to 5 percent of the weight of the fish. The temperature of the domestication phase is controlled at 30°C ± 1°C, and changes are made after the start of the experiment.

The experiment is divided into 5 groups according to the temperature stimulation: 30°C, 32°C, 34°C, 36°C and 38°C. Each barrel contains 12 fish. The 30°C group kept the temperature unchanged as the 30°C as a control group; the rest controlled the temperature rise at a speed of 0.5°C/h (the error is within ±0.1°C); the stimulus total duration is controlled at 8 days; and the arrival time at the same speed controls the temperature drop to 30°C. Continue feeding for 10 weeks at 30°C after the end of the high-temperature stimulation experiment.

### 2.3 Sample collection and analysis

At the end of the high-temperature experiment, a 24-h fasting process was carried out, with a total of 45 fish randomly selected from the three nets of each experimental group, 3 fish selected for sampling in each net cage. The batch was rapidly anesthetized with MS-222 solution at a concentration of 100 mg/L (Shanghai Experimental Reactor Co., Ltd., Shanghai, China), and tissue samples were obtained.

At the end of the feeding experiment, three fish were selected again from each net box using the same method, and the fish were rapidly anesthetized, precisely measuring the weight and length of each fish.

Three fish are selected from each net box, and their intestinal tissues are harvested. They are first cleaned with a cold phosphate buffer solution (PBS) and then cut into small pieces of intestinal tissue. The remainder is ground in cold saline water (at a ratio of 1:10 w/v) for further treatment. After 10 min of centrifugation at a speed of 4,000 rpm at 4°C, the supernate is collected, which is then used to measure the activity of digestive enzymes (including AMS, LPS and TRY), and the measurement is done through the commercial reagent box provided by the Nanjing Institute of Health and Biotechnology. Their liver tissue is collected according to the same processing method and is divided into three parts. First, use cold phosphate buffer solution (PBS) cleaning; the first part retains a 2–3 cm length; a more complete-shaped part of the tissue is fixed in polymorphic formaldehyde for 24 h; the tissues were embedded in paraffin, and the wax blocks are cut into 3 μm thick; Hematoxylin-Eosin dye (H&E dyeing); and finally, dehydration and sealing are performed. The vertical Ni-E microscope of the Nikon Ds-Ri2 camera (Nikon, Tokyo, Japan) was used for microscopic examination, and the slice images were collected. Half of the second part of the liver tissue is used to measure the activity of oxidative stress-related enzymes (including SOD, peroxide, and hydroxide) and LZM using the same method as the intestine using physiological salt water grinding, centrifugation, and collecting supernate using commercial reagent boxes (provided by the Nanjing Institute of Biotechnology). The third part is ground with liquid nitrogen freezing, and the total RNA is obtained through Trizol reagent (Ambion, Texas, United States) from lysed tissue cells. The Agilent 2100 bioanalyzers (Agilent Technologies, Santa Clara, CA, United States) and NanoDrop 2000 (Thermo Scientific, Wilmington, DE, United States) are used to evaluate RNA integrity and quality. Following the detailed steps in the instructions that come with the cDNA synthesis reagent box (Ambion, Texas, United States), a reverse transcriptase reaction is carried out on total RNA. The gene sequence came from the GenBank database, and Biobio Engineering Co., Ltd. (Shanghai, China) synthesized the qPCR derivatives needed for the experiments (Table 1). The SYBR Green PCR Master Mix reagent box (TaKaRa, Kusatsu, Japan) is used for real-time PCR (qPCR) with the ROCHE Light Cycler 96 real-time system (Roche, Switzerland). The amplification conditions included preheating at 95°C for 120 s and 45 PCR cycles (94°C for 10 s; 62°C 30 s; 72°C 10 s). Each reaction system contains 5.2 μL of SYBR mixture, 2.8 μL of ultrapure water, 0.5 μL of forward primer, 0.5 μL of reverse primer, and 1 μL of cDNA as templates. In this experimental step, β-actin is used as an internal reference gene gene to calibrate the expression data of the target gene, ensuring the accuracy and reliability of the analysis results. The relative abundance of target gene mRNA was calculated by R = 2^−ΔΔCT^, and each liver sample was repeated three times to ensure the stability of the experimental results.

**TABLE 1:** Primer sequence for qPCR.

Genes	Primers (5′–3′)	Accession No
*IL-1β*	F: 5′ AGCACTGAAGCCAGACCA 3′	XM_020585780.1
	R: 5′ GAA​CAG​AAA​TCG​CAC​CAT​A 3′	
*TLR1*	F: 5′AAC​CGG​GCT​GCT​TTT​ATG​GA3′	XM_020624433.1
	R: 5′TGG​GCT​TCA​TTG​TCT​GCC​TTT3′	
*irak3*	F: 5′ GAC​CAA​GCA​TGG​AGA​AGG​TAC​T 3′	XM_020601231.1
	R: 5′ GTA​TGG​ACA​ACA​GGG​GGC​TC 3′	
*C3*	F: 5’ GAC​TGT​TGT​TTG​GAC​GGC​AT 3′	XM_020587458.1
	R: 5′ GTC​ATC​TTC​CTC​ACT​CCG​ACC 3′	
*TNF-α*	F: 5′ TGACAAACCCGCAGAAGA 3′	XM_020621700.1
	R: 5′ CGT​AAA​CCT​CCA​GGT​AAT​CG 3′	
*HSP90α*	F: 5′ GTA​GGC​TGG​GCT​TTC​TCG​AAT 3′	XM_020603713.1
	R: 5′ GTG​TGC​TTC​AGG​CAT​CTC​TAT​C 3′	
*β-actin*	F: 5′ GCG​TGA​CAT​CAA​GGA​GAA​GC 3′	XM_020621264.1
	R: 5′ CTC​TGG​GCA​ACG​GAA​CCT​CT 3′	

For intestinal microbiome analysis, DNA from the sample was extracted from the DNeasyPowerSoil reagent box (QIAGEN, Hilden, Germany), and the DNA concentration was detected by NanoDrop 2000 (Thermo Fisher Scientific, Waltham, MA, United States). The 16S rRNA gene sequencing was completed on the NovaSeq 6000 platform of Ilumina (Santiago Illumina, California, United States; Shanghai OE Biotechnology, China).

### 2.4 Statistical analysis

The data is computed using Microsoft Excel, evaluated with SPSS 22.0 program (IBM Company, NY, United States) for statistical analysis, and GraphPad Prism version 9.5 is utilized for graphing. Significant differences in groups were assessed using single-factor differential analysis (ANOVA) and graphical testing. All information is displayed as the average standard deviation (SD), where *p* < 0.05 is considered statistically significant.

## 3 Results

### 3.1 Basic growth parameters

Table 2 displays the impact of high temperature stimulation on the growth rate of *M. albus*. CF, HSI, and VSI did not exhibit a statistically significant difference (*p* > 0.05). The FCR, FBW, WGR, and SGR of *M. albus* larvae at 38°C differed substantially from the other four groups (*p* < 0.05). Compared with the 30°C group, the survival rate of other groups tends to increase (*p* < 0.1). When the high temperature stimulation reached 34°C, the growth performance of *M. albus* reached the highest level. When the high temperature stimulation reached 36°C, the growth performance of *M. albus* were inhibited.

**TABLE 2:** Effect of high temperature stimulation on growth performance of *M. albus* after 10 weeks.

Parameters	Temperature level					*p*-value
	30°C	32°C	34°C	36°C	38°C	ANOVA
IBW^a^	12.73 ± 0.81	12.62 ± 0.55	12.44 ± 0.63	12.2 ± 0.57	12.69 ± 0.48	0.366
FBW^b^	36.04 ± 0.47^b^	36.07 ± 1.23^b^	38.08 ± 1.73^a^	34.54 ± 1.13^b^	31.45 ± 1.12^c^	< 0.001
SR^c^	0.78 ± 0.05	0.81 ± 0.1	0.83 ± 0.08	0.72 ± 0.17	0.58 ± 0.09	0.097
WGR^d^	184.09 ± 16.57^b^	186.38 ± 18.88^ab^	206.48 ± 16.72^a^	183.69 ± 16.83^b^	148.19 ± 12.56^c^	< 0.001
SGR^e^	1.49 ± 0.08^a^	1.5 ± 0.09^a^	1.6 ± 0.08^a^	1.49 ± 0.09^a^	1.3 ± 0.07^b^	< 0.001
FCR^f^	2.25 ± 0.06^b^	2.25 ± 0.14^b^	2.06 ± 0.14^c^	2.36 ± 0.15^b^	2.81 ± 0.17^a^	0.039
CF^g^	7.89 ± 1.19	7.82 ± 1.05	7.52 ± 1.64	6.31 ± 1.33	6.77 ± 0.88	0.069
HSI^h^	1.46 ± 0.59	2.36 ± 0.89	1.76 ± 0.41	1.81 ± 0.78	2.37 ± 1.08	0.181
VSI^i^	5.95 ± 1.13	7.81 ± 1.99	6.2 ± 2.89	5.51 ± 1.39	6.36 ± 2.20	0.157

Note: Value are presented as means ± SD (standard deviation). Different lowercase letters indicate significance difference among the groups (*p* < 0.05).

^a^ IBW, initial body weight (g).

^b^ FBW, final body weight (g).

^c^ SR, survival rate (%) = 100 × final number of fish/initial number of fish.

^d^ WGR, weight gain rate (%) = 100 × (final body weight—initial body weight)/initial body eight.

^e^ SGR, specific growth rate (%/d) = 100 × (ln FBW—ln IBW)/70 days.

^f^ FCR, feed conversion rate = feed intake/(final body weight—initial body weight).

^g^ CF, condition factor (%) = 100 × (body weight)/(body length)^c^.

^h^ HSI, hepatopancreas index (%) = 100% × (liver weight)/(final body weight).

^i^ VSI, viscerosomatic index (%) = 100% × (viscera weight)/(final body weight).

### 3.2 Intestinal digestive enzyme activity

**FIGURE 1:**
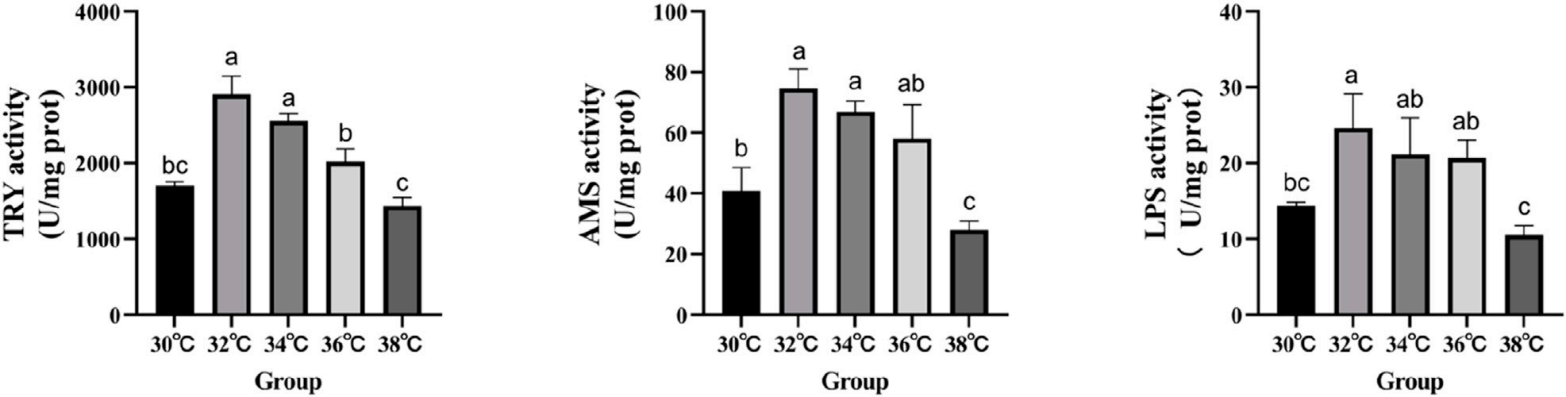
Effect of high temperature stimulation on digestive enzyme activity in the intestine of *M. albus*.

The levels of digestive enzyme activity are measured in order to conduct an in-depth analysis of the effects that high-temperature stimulation has on the levels of nutritional absorption in the intestinal tract (Figure 1). When the stimulation temperature reaches 34°C, the activity of AMS, LPS and TRY is at its peak.

### 3.3 Liver antioxidant and antibacterial enzymes activity

**FIGURE 2:**
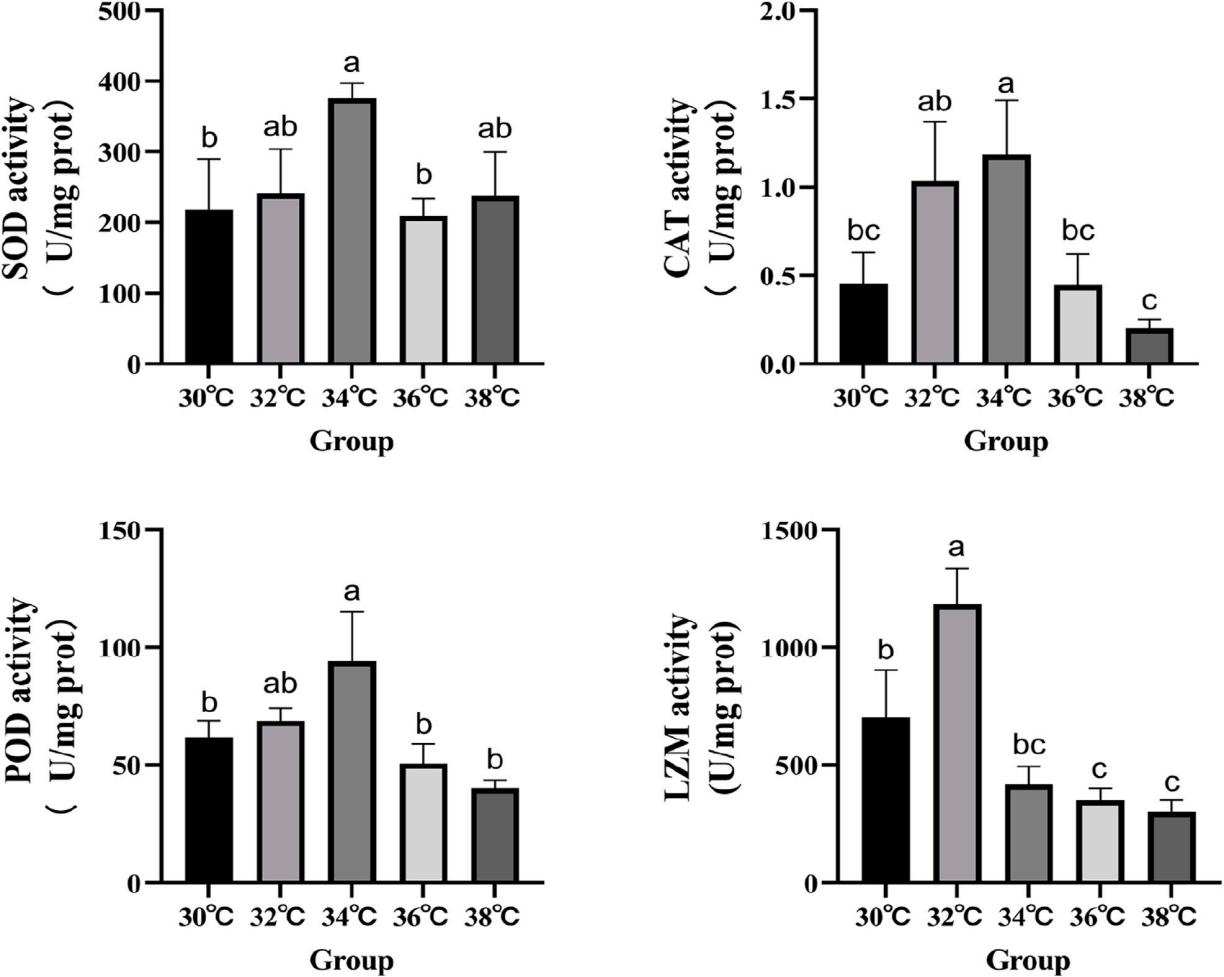
Effects of high temperature stimulation on activities of antioxidant enzymes and lysozyme in the liver of *M. albus*.

The levels of antioxidant activity were measured in order to investigate the effects of high-temperature stimulation on oxidative stress and immunity (Figure 2). The study demonstrated that the levels of SOD, CAT, and POD activity in the experimental group reached their highest point (*p* < 0.05) when exposed to high-temperature stimulation of 34°C. LZM’s performance significantly increased at 32°C compared to the 30°C group, reaching statistical significance (*p* < 0.05).

### 3.4 Liver morphology

**FIGURE 3:**
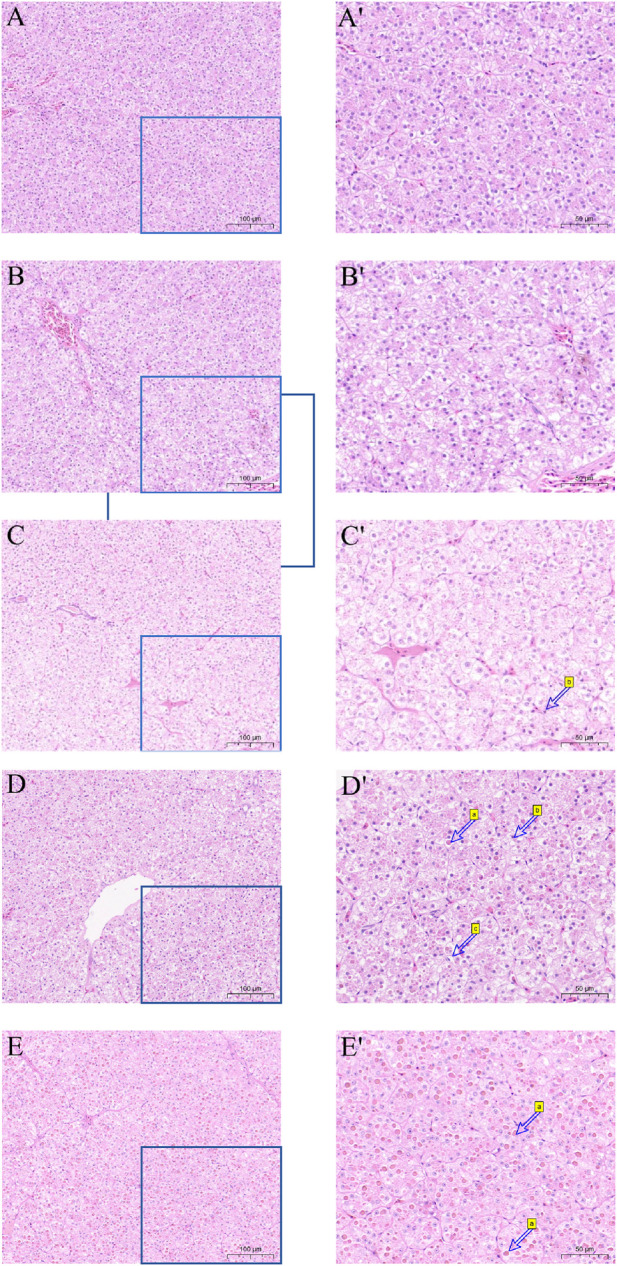
Effect of high temperature stress on liver morphology (× 100 and × 200) of *M. albus*. **(A,A′)** 30°C **(B,B′)** 32°C **(C,C′)** 34°C **(D,D′)** 36°C **(E,E′)** 38°C, a cell necrosis, b nucleus condensation and c vacuole.

Hematoxylin and eosin (H&E) were utilized to stain the liver tissue sections, and the outcome is displayed in Figure 3. The liver cell shape of the 30°C group is normal, the arrangement is smooth, the boundaries are clear, and the cell mass is uniform. When the temperature stimulus reaches 36°C, the liver cells are markedly damaged, including foam deformation, cell death, cell shape blurred, and nucleic solidificaion. As the degree of thermal suppression increases, tissue damage becomes more noticeable.

### 3.5 Expression of genes associated to the hepatic immune system

**FIGURE 4:**
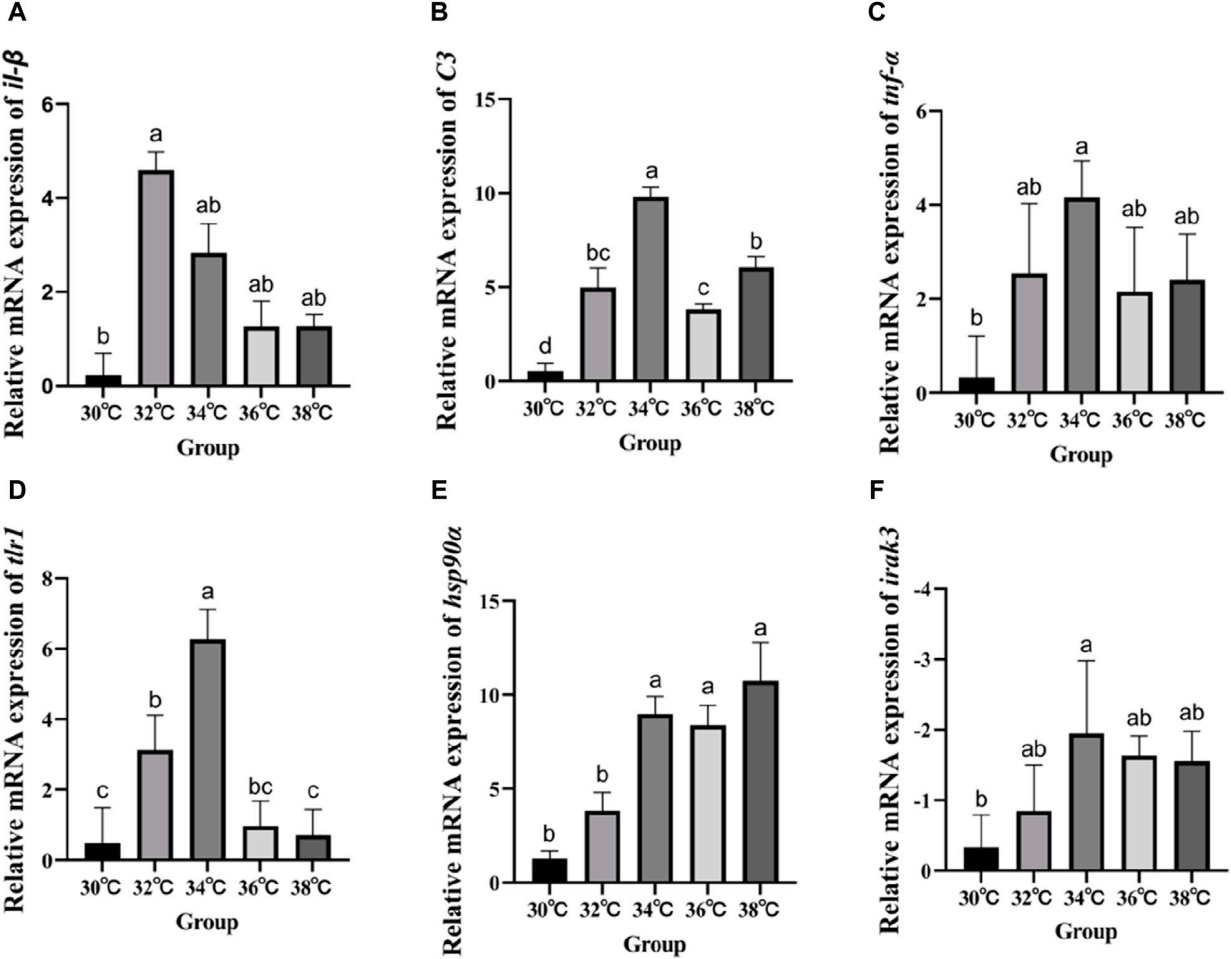
Effect of high temperature stimulation on mRNA expression level in liver of *M. albus*. **(A)** *IL-1β*: Interleukin 1β, **(B)** *C3*: complement component 3, **(C)** *TNF-α*: tumor necrosis factor α, **(D)** *tlr1*:Toll-like receptor 1, **(E)** *hsp90α*: heat shock protein 90α, and **(F)** *irak3*: IL-1 receptor-associated kinase 3.

Figure 4 displays the variations in the expression of *IRAK3*, *C3*, *TNF-α*, *IL-β*, *Hsp90*, and *TLR1* in the liver of *M. albus* larvae under various temperature conditions. The gene expression of *Hsp90* significantly increased when the temperature stimulus changed in comparison to the 30°C group. Genes including *C3*, *TNF-α*, *IL-β*, and *TLR1* show a similar pattern of expression following exposure to high temperatures, initially increasing and then reducing before peaking at 34°C. In contrast, the *IRAK3* gene exhibits an opposite trend, initially decreasing in expression before increasing (*p* < 0.05).

### 3.6 Intestinal microbial diversity index calculated on the basis of 16S rDNA gene sequence

**FIGURE 5:**
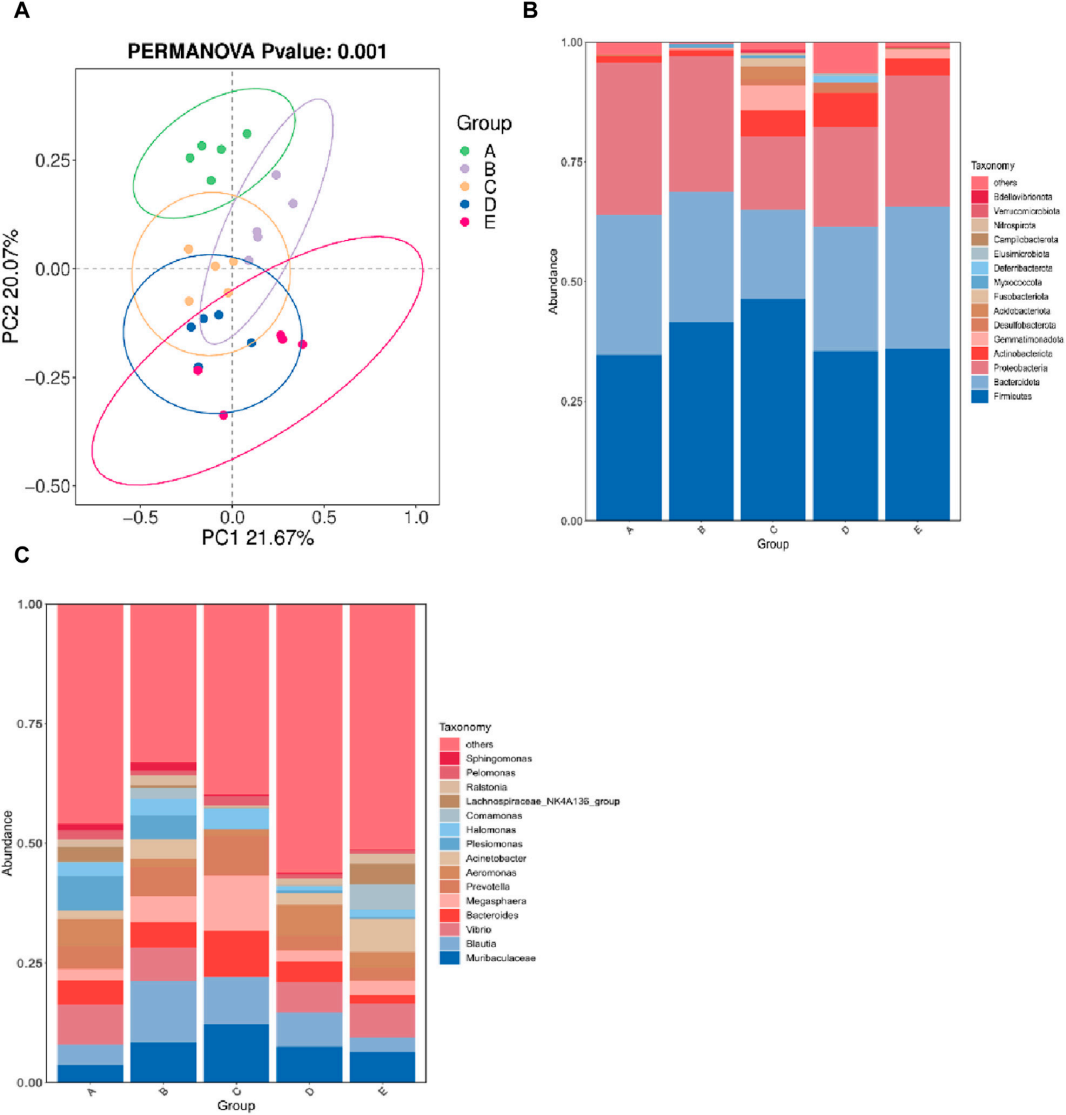
Effect of high temperature stimulation on intestinal flora of *M. albus*. Group A: 30°C group; Group B: 32°C group; Group C: 34°C group; Group D: 36°C group; Group E: 38°C group. **(A)** Principal Coordinate Analysis (PCoA) of the effect of high temperature stimulation on the diversity index of *M. albus* microbial community. **(B)** Effect of high temperature stimulation on phylum of microbiota diversity index in *M. albus*. **(C)** Effect of high temperature stimulation on genus of microbiota diversity index in *M. albus*.

The α diversity index reflects the abundance and diversity of intestinal microbial populations, as shown in Table 3. All groups had 100% goods coverage of commodities, suggesting that the sequencing depth adequately represented all species in the sample. Compared with the 30°C group, the indexes of observed species, Shannon and Chao1 in the 34°C group increased significantly (*p* < 0.05), and were also better than those in other treatment groups, and the change trend of these diversity indexes was the same. The main coordinate analysis of all groups (PCoA) divides samples into groups on the coordinate chart, showing how high temperatures change the make-up of microbes in the gut. PCoA showed that the intestinal microflora of the other group was separated from that of the 30°C group. The results showed that the intestinal microflora showed significant differences in β diversity after being stimulated by high temperature (Figure 5A). The abundance of the intestinal microbial communities of the *M. albus* samples surveyed is shown in Figure 5B, with *Proteus*, *Firmicutes*, *Bacteroides* and *Actinomycetes* as the main phylums. The abundance of *Proteobacteria* initially declined and then increased compared to the 30°C group, whereas the abundance of *Bacteroides* increased. *Firmicutes* initially developed and subsequently declined. To further study the specific differences in microbiomes, Figure 5C shows the abundance of microbiomes in the first 15 genus. In the other group, *Bacteroides* and *Muribaculaceae* was increasing compared to the 30°C group, while the *Vibrio*, *Aeromonas* were decreasing.

**TABLE 3:** Effect of temperature stimulation level on α diversity of intestinal microorganisms in *M. albus*.

Parameters	Temperature level					*p*-value
	30°C	32°C	34°C	36°C	38°C	ANOVA
Goods coverage	1.00 ± 0.00	1.00 ± 0.00	1.00 ± 0.00	1.00 ± 0.00	1.00 ± 0.00	—
Shannon	3.31 ± 0.64^b^	4.03 ± 0.74^ab^	5.28 ± 1.57^a^	3.48 ± 0.32^b^	4.36 ± 0.25^ab^	0.011
Observed species	82.14 ± 18.65^ab^	78.04 ± 15.19^ab^	162.32 ± 77.20^a^	75.48 ± 11.36^b^	71.22 ± 30.01^b^	0.022
Simpson	0.87 ± 0.07	0.88 ± 0.05	0.94 ± 0.03	0.83 ± 0.04	0.86 ± 0.08	0.083
Chao1	84.47 ± 20.47^ab^	97.2 ± 24.37^ab^	153.03 ± 87.74^a^	73.85 ± 10.79^b^	65.22 ± 21.02^b^	0.033

Note: Value are presented as means ± SD (standard deviation). Different lowercase letters indicate significance difference among the groups (*p* < 0.05).

## 4 Discussions

Generally, there is an optimum temperature for the growth of fish. If the temperature is too high, it will reduce growth performance and even lead to death (Islam et al., 2022). The findings of this study demonstrated that, after acute temperature stress, *M. albus*’s FBW, WGR, SGR, and FCR exhibited a significant tendency of first increasing and then dropping with temperature as compared to the 30°C group. Similar to studies on other fish, this demonstrates that suitable temperature stress can enhance *M. albus* growth performance and is a driving factor in regulating its growth performance (Pang et al., 2016; Ashaf-Ud-Doulah et al., 2020; Huss et al., 2021; Zhang et al., 2021). It is worth noting that, similar to this study, these results are aimed at the young fish of the research object. In fish with variable temperatures, the environmental temperature will affect the growth performance by affecting the fish’s food intake, digestion, absorption, and metabolic activities (Fu et al., 2018; Volkoff and Rønnestad, 2020), and an appropriate temperature increase will improve these performances (Takasuka and Aoki, 2006; Sotoyama et al., 2018; Li et al., 2023). The growth performance in this experiment indicated that 32°C–34°C was the ideal temperature for *M. albus* under high temperature stress, which was in line with our earlier hypothesis. In the long-term feeding procedure that followed, *M. albus* under this temperature stress exhibited good activity and food intake. The growth performance of *M. albus* in the follow-up feeding procedure was, however, inhibited to various degrees in the two groups of 36°C and 38°C due to the increase in stress temperature. It is speculated that more energy should be allocated and the cell structure damage caused by temperature stress should be repaired, which showed lower activity and food intake compared with the 32°C and 34°C groups in the follow-up feeding process. It’s interesting that the development of gonads got a lot better in the 32°C and 34°C groups compared to the 30°C group. This suggests that the right amount of temperature stress may help *M. albus* reproduce, but more research is needed to be sure.

As is well knowledge, fish growth and development performance are directly correlated with the activity of their digestive enzymes. AMS, TRY, and LPS are three common digestive enzymes that can hydrolyze carbohydrates, proteins, and lipids, respectively, so as to obtain the energy needed for maintaining life activities, growth, and reproduction. Our findings indicate that following high temperature stress, the 34°C group’s various enzymes activity were significantly higher than those of the 30°C group. They also did better than other treatment groups. This more intuitively explains why the growth performance of *M. albus* in the 34°C group was significantly improved, and similar conclusion were obtained in other fish (Qiang et al., 2017; Liu et al., 2023).

As the most vital organ in *M. albus*, the liver is directly communicates with the heart, intestine and gallbladder, related to growth and development, substance metabolism, detoxification, and hematopoiesis and can also reflect the physiological, pathological, and nutritional status of *M. albus*. Excessive temperature can also cause structural damage to fish, especially the liver, which has been confirmed in many species (Dettleff et al., 2022; E. Liu et al., 2022; Zhao et al., 2022). Excessive temperature will also stimulate the production of endogenous reactive oxygen species (ROS) (Wiens et al., 2017). The stability of the liver’s structure is directly linked to its function as a site for the production of detoxified reactive oxygen species; yet, an excess of ROS can impact the liver’s structure by changing the condition of the hepatocytes (Qin et al., 2023). The results from the 36°C and 38°C groups support the conclusion that high temperature stress will cause irreversible damage to the liver of *M. albus*, leading to significant impacts on its growth performance. High temperature has been proven to damage the livers of fish, especially cold-water fish (Ma et al., 2023).

The health of the liver is also very important for the normal operation of the immune system of *M. albus*, but the damage to the liver structure will have a serious impact on the immunological system of fish (Peng et al., 2022). SOD and CAT are considered the important first lines of defense against ROS because they can transform ROS into harmless metabolites, thus effectively preventing the accumulation of ROS (Yang et al., 2023). LZM is a crucial defensive molecule of the fish natural immune system that can take part in the immunological response to bacterial attack and heat stress (Saurabh and Sahoo, 2008; C. Zhang et al., 2018). Our research shows that, compared with the 30°C group, the activity of these antioxidant enzymes can be significantly improved at 34°C after high temperature stress, and it is more stable, but it tends to decrease at higher temperatures. This shows that individuals can eliminate the damage of peroxides and superoxide radicals by enhancing the activity of antioxidant enzymes in the culture of *M. albus* under a high temperature stress of 34°C, so as to improve the health status and growth and metabolic performance of the liver. The opposite effect will occur at too high a temperature. The expression of complement and heat shock proteins is beneficial to the improvement of immunity. *C3* complement is one of the key proteins for fish to adapt to temperature change, and it is expressed in immune-related tissues such as the gills, blood, and liver (Qi et al., 2011; Xu et al., 2018). High temperature stress will strengthen the expression of the *C3* complement gene in fish, thus affecting its immunity (Najafpour et al., 2020), which is proved by the research findings of carp (Dietrich et al., 2018). Heat shock proteins, including *HSP90α*, also participate in the immune response of fish (Wan et al., 2020). In some studies, the transcription level of HSP mRNA is regarded as a molecular biomarker of liver histological disorder caused by high temperature stress (Li et al., 2019). Fish immune systems can sense heat shock proteins and interact with pattern recognition receptors to activate antigens (Baharloei et al., 2021), thus regulating the immunological system to enhance the ability of fish to resist bacterial and viral infections (Jeyachandran et al., 2023). Therefore, the expression of the *HSP90α* gene in response to temperature changes may affect immune ability by regulating protein deposition and participating in innate and adaptive cellular immunity (Hoter et al., 2018). The toll-like receptor (*TLR1*) signal system plays a crucial role in the natural immunity of *M. albus TLR1* is a significant contributor to the natural immune response (Arancibia et al., 2007), and *TLR1*s are the fastest natural immune receptors to respond to fish infection (Li et al., 2017). Fish-specific *TLR1* acts a key role in the natural immune response of teleost (Nie et al., 2018; Shan et al., 2021). The *TLR1*-mediated signal transduction pathway is complex, involving a variety of proteins, including *IL-1β*, *TNF-α*, and *IRAK* (Fitzgerald and Kagan, 2020). High temperatures can stimulate the transcription of *IL-1β* and *TNF-α*, thus affecting the Toll-like receptor-mediated immunity in fish (Basu et al., 2012; Zou and Secombes, 2016). *IL-1β* may induce other cells to produce pro-inflammatory molecules. *TNF-α* triggers apoptosis and activates endothelium cells, hence enhancing the immunological response of fish against bacterial and viral infections (Roca et al., 2008) and has a function in the immune response against bacterial and viral pathogens. *IRAK*, which includes *IRAK3*, is responsible for facilitating the connection of various *IL-1* signaling pathways and increasing the release of cytokines that promote inflammation (Zheng and He, 2022). These cytokines can stop *TLR1* signals from activating (Rebl and Goldammer, 2018), lowering the immune response. The decrease in *IRAK3* gene expression and the increase in *TNF-α*, *IL-β*, and *TLR1* gene expression indicate that high temperature stress activates this signaling pathway. Fish benefit from even a temporary boost in immunity as it helps them adjust to their new immunological environment, even though the effects of high temperature stimulation eventually diminish (Wang et al., 2023).

An increasing amount of studies indicates that fish growth and development, as well as intestinal health, are influenced by the makeup of intestinal bacteria (El-Saadony et al., 2021; Y. Liu et al., 2022; López Nadal et al., 2020). The predominant bacterial species in the gut of *M. albus* were discovered to be Firmicutes, Bacteroidota, and Proteobacteria. This finding is in line with studies on the intestinal flora composition of numerous other aquatic creatures. Following a period of high temperature stress, Firmicute abundance first declined and subsequently increased as temperature rose. Firmicutes are thought to contribute to intestinal health because of their capacity to interact with intestinal mucosa and ferment dietary fiber (Sun et al., 2023). Furthermore, research indicates that animal obesity and fat accumulation may be associated with the ratio of Firmicutes to Bacteroidota (Ley et al., 2006; Mariat et al., 2009; Sun et al., 2023). The findings of our investigation are comparable. Following a stress of high temperature, 34°C Group is able to demonstrate greater growth performance when compared to the control group. Muribaculaceae perform a number of crucial functions at the genus level in the breakdown of complex polysaccharides. Numerous multifunctional carbohydrate active enzymes, found in humans, mice, and other animals, are present in the studied genome (Ormerod et al., 2016). These enzymes are similar to the trend of improvement in perch’s digestive function (Lin et al., 2023). Muribaculaceae contributes to the breakdown of complex carbohydrates, which can result in the production of acetic and propionic acids, and is positively correlated with the intestinal mucus layer’s barrier function (Lagkouvardos et al., 2019). It’s interesting to note that our findings indicate that 34°C Group has a substantially higher Megasphaera abundance than the other groups. The presence of Megasphaera in the body is associated with increased levels of immune genes in the gut and blood nutritional indicators (Brown et al., 2019), suggesting that it may be a probiotic. It is a bacteria that can convert many kinds of short-chain fatty acids (SCFA) from lactic acid, including acetate, propionate, butyrate, and valerate (J. Zhang et al., 2018). Since they are the host’s energy source, these SCFA are crucial for intestinal health. As probiotics, Megasphaera has been shown to benefit pig and rodent digestive systems (Hashizume et al., 2003; Xie et al., 2017). Thus, we hypothesize that *M. albus*’s intestinal microorganisms can be reshaped and that the quantity of some bacteria can be markedly increased following the temperature stress of 34°C. The beneficial effects of these bacteria on intestinal health and obesity in animals have been demonstrated by science. This demonstrates that *M. albus* growth performance can be enhanced following appropriate temperature stress.

## 5 Conclusion

The high temperature stress of 34°C can stimulate the growth rate of *M. albus* larvae and when the temperature exceeds 36°C, it has a negative effect on the body, cause cell damage in the liver of *M. albus* and stimulate the immune system. The activities of digestive enzymes such as AMS, LPS and TRY in the intestine increased. The activities of SOD, POD and LZM will increase after high temperature stress, and the expression of *HSP90α*, *C3* and other immune-related protein synthesis genes will be enhanced. The expression of *TLR1*, *IL-1β*, *TNF-α* and other genes increased under high temperature stress, while the expression of *IRAK3* gene decreased, and the *TLR1* signaling pathway was activated. High temperature stimulation will also change the diversity of intestinal microorganisms and affect the nutritional absorption of the intestine. The changes in the physiological status and gene expression of *M. albus* after being stimulated by high temperatures below 36°C, will generally have beneficial effects on immunity and growth rate. Generally speaking, when the temperature rises to 34°C, it is beneficial to the growth and immunity of the cultured objects. When it exceeds this limit, the body of *M. albus* is damaged, but there is no significant difference in its mortality.

## Data Availability

The raw data supporting the conclusions of this article will be made available by the authors, without undue reservation.
